# Neocortex and Allocortex Respond Differentially to Cellular Stress *In Vitro* and Aging *In Vivo*

**DOI:** 10.1371/journal.pone.0058596

**Published:** 2013-03-11

**Authors:** Jessica M. Posimo, Amanda M. Titler, Hailey J. H. Choi, Ajay S. Unnithan, Rehana K. Leak

**Affiliations:** Division of Pharmaceutical Sciences, Mylan School of Pharmacy, Duquesne University, Pittsburgh, Pennsylvania, United States of America; University of Pittsburgh, United States of America

## Abstract

In Parkinson’s and Alzheimer’s diseases, the allocortex accumulates aggregated proteins such as synuclein and tau well before neocortex. We present a new high-throughput model of this topographic difference by microdissecting neocortex and allocortex from the postnatal rat and treating them in parallel fashion with toxins. Allocortical cultures were more vulnerable to low concentrations of the proteasome inhibitors MG132 and PSI but not the oxidative poison H_2_O_2_. The proteasome appeared to be more impaired in allocortex because MG132 raised ubiquitin-conjugated proteins and lowered proteasome activity in allocortex more than neocortex. Allocortex cultures were more vulnerable to MG132 despite greater MG132-induced rises in heat shock protein 70, heme oxygenase 1, and catalase. Proteasome subunits PA700 and PA28 were also higher in allocortex cultures, suggesting compensatory adaptations to greater proteasome impairment. Glutathione and ceruloplasmin were not robustly MG132-responsive and were basally higher in neocortical cultures. Notably, neocortex cultures became as vulnerable to MG132 as allocortex when glutathione synthesis or autophagic defenses were inhibited. Conversely, the glutathione precursor N-acetyl cysteine rendered allocortex resilient to MG132. Glutathione and ceruloplasmin levels were then examined *in vivo* as a function of age because aging is a natural model of proteasome inhibition and oxidative stress. Allocortical glutathione levels rose linearly with age but were similar to neocortex in whole tissue lysates. In contrast, ceruloplasmin levels were strikingly higher in neocortex at all ages and rose linearly until middle age. PA28 levels rose with age and were higher in allocortex *in vivo*, also paralleling *in vitro* data. These neo- and allocortical differences have implications for the many studies that treat the telencephalic mantle as a single unit. Our observations suggest that the topographic progression of protein aggregations through the cerebrum may reflect differential responses to low level protein-misfolding stress but also reveal impressive compensatory adaptations in allocortex.

## Introduction

Neurodegenerative diseases are often described as proteinopathies or diseases of protein homeostasis (proteostasis), because the rate of formation of misfolded proteins is out of balance with their clearance [1]–[8]. For example, aggregations of α-synuclein and tau are considered hallmarks of Parkinson’s and Alzheimer’s diseases, respectively. Regional variations in the distribution of aggregated proteins across the brain are used to stage these illnesses according to Heiko Braak’s criteria [9]–[12]. Such staging protocols assume that topographical variations in misfolded proteins reflect the progression of initially prodromal and subsequently symptomatic clinical syndromes [13]–[16]. Although this assumption has fallen under some scrutiny, Alzheimer’s and Parkinson’s staging criteria reveal a striking congruence in topographical vulnerability to proteotoxic stress in the cortex. That is, the temporal allocortex appears to be the nucleation site for cortical spread of protein misfolding in both diseases, whereas the 6-layered neocortex (also called isocortex) is affected last [17], [18]. More specifically, the pre- and post-central gyri encompassing primary motor and sensory regions of the frontal and parietal neocortical lobes are the last to surrender to protein aggregations. In addition to Parkinson’s and Alzheimer’s diseases, this conspicuous staging of cortical vulnerability is also apparent in several other brain disorders, including Huntington’s, Pick’s, and argyrophilic grain diseases [9], [18]–[22].

Three decades ago, Brun and Englund described a regional pattern of degeneration in Alzheimer’s such that the entorhinal cortex in medial temporobasal areas was severely affected relative to the central lobe that housed the pre- and postcentral gyri [23]. Indeed, sparing of this central lobe was noted by Alois Alzheimer himself as well as several authors in the 50s, 60s, and 70s (discussed by Brun and Englund). In these studies the frontal gyri were also affected late in the disease, albeit before the pre- and postcentral gyri. In contrast to the relative sparing of these latter regions, investigators have noted the near-complete collapse of the vulnerable temporal lobe. This histological pattern correlates with early reductions in physiological function, as metabolism and perfusion are reduced in the medial temporal lobe in both mild cognitive impairment and early Alzheimer’s disease [24]–[27]. Furthermore, the neuropathological staging of Alzheimer’s disease is linearly correlated with intellectual status [28]. More specifically, both memory and executive function are correlated with neuronal loss in the entorhinal cortex [29]–[31]. It should be noted here that the amyloid burden in Alzheimer’s disease actually begins in neocortex [32], [33]. However, cerebral atrophy follows the topography of tau pathology instead [34]–[36]. Furthermore, total plaque numbers do not correlate with the disease severity [37] or with loss of neurons [30].

The high risk of developing dementia in Parkinson’s disease is similarly associated with cortical pathology [18], [38]–[41]. Synuclein inclusions encroach upon cortex in Braak stages 5 and 6 and are linked to cognitive dysfunction [42]. Several reports note that cognitive impairment correlates with the presence of cortical Lewy bodies, specifically those in the entorhinal, frontal, and cingulate cortex [39], [43]–[46]. However, in contrast to the vulnerable temporal lobe, only few Lewy bodies are present in the pre- and postcentral gyri in Parkinson’s postmortem tissue [43], [47]. As in Alzheimer’s disease, there is prominent atrophy of the medial temporal lobe in Parkinson’s disease [48], [49]. In particular, the atrophy in the allocortical hippocampus correlates with patients’ verbal memory scores [50], [51]. As a result, Parkinson’s disease is becoming increasingly recognized as being much more than a movement disorder; patients are known to suffer from a broad constellation of non-motor symptoms that do not respond well to dopaminergic therapy [52]. Thus, it is important to study extranigral pathology in the context of insults relevant to Parkinson’s disease.

The goal of the present study was to develop a high-throughput model of allocortical versus neocortical degeneration that recapitulates the topography of vulnerability in humans. For studies of regional differences in human diseases, *in vitro* models harvested from rodent brains are not entirely unprecedented, as similar studies to model loss of hippocampal subregions in Alzheimer’s have already met with success [53]. Although it is the mesocortex (comprised of periallo- and proneocortex, the transentorhinal region of the rhinal sulcus) as well as the allocortex (comprised of paleo- and archicortex, ventral to the rhinal sulcus) that Braak describes as the Achilles’ heel of the telencephalic edifice [17], [18], the transentorhinal mesocortex is primate-specific, and so we focus only on allocortex in our rodent model. Our neocortical microdissections are centered in the primary motor and sensory cortex whereas our allocortical dissections are centered in piriform and entorhinal cortices at the opposite pole of the postnatal brain. We excluded hippocampus from our allocortical dissections because it is so structurally dissimilar to neocortex. Piriform cortex is comprised of 3 layers, meeting the definition of trilaminar paleocortex, or allocortex. By strict anatomical criteria, entorhinal cortex is actually a transitional zone and composed of more than 3 layers, but it is more closely associated with the ancient archicortex and so is classified as allocortex [18]. In contrast, the phylogenetically more modern neocortex is comprised of 6 layers and is much more heavily myelinated. Because of this difference in ensheathment by oligodendrocytes, Braak hypothesizes that allocortex suffers higher rates of metabolism during neuronal firing and consequent oxidative stress [17]. In addition, Braak hypothesizes that the staged involvement of allocortex followed by neocortex can be explained by an insult travelling through the brain in transneuronal fashion, affecting different neuronal subgroups like a falling row of dominoes [17]. In the present study, we hypothesize an easy-to-test alternative to Braak’s hypothesis that might act in concert with cell-to-cell transmissibility: differences in neuronal resilience to cellular stress may also partly determine the sequence in which brain regions are collared into neurodegenerative disease.

To tackle this hypothesis, we developed an *in vitro* model in which parallel primary cultures of neo- and allocortex from the same rat pup brains were treated with two types of toxins relevant to neurodegeneration. First, the proteasome inhibitors MG132 and PSI were chosen to elicit proteotoxic stress, as inhibition of the ubiquitin-proteasome system is evident in both Alzheimer’s and Parkinson’s disease [54]–[58]. Second, the commonly used oxidative poison H_2_O_2_ was chosen because indices of oxidative stress are also plentiful in postmortem tissue in both Alzheimer’s and Parkinson’s disease [59]–[64]. We contrasted the levels of proteasome activity and ubiquitin-conjugated proteins in neo- and allocortical cultures, and investigated the potential interaction between proteasome inhibition and autophagy. In order to gauge the stress-sensitivity of neo- and allocortex, we assessed changes in antioxidant and chaperone proteins in response to MG132. The mechanistic role of the ubiquitous tripeptide glutathione was scrutinized, as both Parkinson’s and Alzheimer’s diseases are characterized by low levels of this critical antioxidant early in the course of the disease process [59], [65]–[70]. N-acetyl cysteine, a glutathione precursor and antioxidant, was examined for its ability to protect allocortical cultures against MG132. N-acetyl cysteine ameliorates cognitive dysfunction in Alzheimer’s disease [71] and is currently undergoing clinical trials in Parkinson’s patients (Clinicaltrials.gov ID: NCT01470027). N-acetyl cysteine is also useful for non-neurodegenerative conditions and offers protection in animal models [72], [73]–[79]. Finally, we examined the impact of *in vivo* aging on glutathione, ceruloplasmin, and proteins related to the proteasome. We hypothesized that neocortex and allocortex exhibit different expression profiles of these molecules as a function of age-related stress. Age is the major risk factor for neurodegenerative diseases. Our second rationale was that the proteasome is increasingly inhibited with aging in the cortex, suggesting that aging is a natural model of proteotoxicity [80]–[82]. Third, aging has long been associated with increased oxidative damage [83].

## Materials and Methods

### Chemicals and Antibodies

Chemicals were purchased from Sigma-Aldrich (St. Louis, MO), unless specified otherwise. Proteasome inhibitors MG132 (EMD Millipore, Billerica MA, Cat. no. 474790) and PSI (Tocris, Minneapolis, MN, Cat. no. 4045) were stored at –20 and –80°C, respectively, as 10 mM stock solutions in dimethyl sulfoxide (DMSO). H_2_O_2_ was purchased from Fisher Scientific (Pittsburgh, PA, Cat. no. H324) and stored at 4°C as an 882 mM stock.

Omission of primary antibodies from the assays always resulted in loss of signal. Primary and secondary antibodies were purchased and used as follows: mouse anti-microtubule associated protein-2 (MAP2, 1∶2000 for immunocytochemistry; Sigma-Aldrich Cat. no. M9942, Lot no. 069K4770), mouse anti-ceruloplasmin (1∶1000 for Western blotting; BD Biosciences, San Diego, CA, Cat. no. 611488, Lot no. 80753), rabbit anti-PA28 (1∶1000 for Western blotting; Calbiochem, San Diego, CA, Cat. no. 539146, Lot no. D00092184), rabbit anti-PA700 10B (1∶1000 for Western blotting; Calbiochem, San Diego, CA, Cat. no. 539147, Lot no. D00110930), rabbit anti-heat shock protein 70 (Hsp70, 1∶5000 for Western blotting; EMD Millipore Cat. no. AB9920), rabbit anti-heme oxygenase 1 (1∶300 for Western blotting; Sigma-Aldrich Cat. no. H4535, Lot no. 081M1122), mouse anti-catalase (1∶5000 for Western blotting; Sigma-Aldrich Cat. no. C0979, clone CAT-505), mouse anti-ubiquitin conjugated proteins (1∶500 for Western blotting; Santa Cruz Biotechnology, Santa Cruz, CA, Cat. no. Absc8017), rabbit anti-glutathione (1∶300 for immunocytochemistry; Millipore Cat. no. AB5010, Lot no. NG1870405), rabbit anti-Beclin 1 (1∶400 for Western blotting; Santa Cruz, CA, Cat. no. SC-11427, Lot no. K2706), rabbit anti-LC3B (1∶1000 for Western blotting; Cell Signaling, Boston, MA, Cat. no. 2775, Lot no. 4), mouse anti-β-actin (1∶40,000 for Western blotting; Sigma-Aldrich Cat. no. A5441, Lot no. 030M4788), mouse anti-α-tubulin (1∶100,000 for Western blotting; Sigma-Aldrich Cat. no. T5168, Lot no. 078K4781), rabbit anti-β-actin (1∶1000 for Western blotting; LI-COR Biosciences, Lincoln, NA, Cat. no. 926-42210, Lot no. C00422-02), rabbit anti-GAPDH (1∶10,000 for Western blotting; Cell Signaling Cat no. 2118, Lot no. 8), infrared 800 nm donkey or goat anti-mouse IgG (1∶2000 for immunocytochemistry; 1∶10,000 for Western blotting; LI-COR Biosciences Cat. nos. 926-32212 and 926-32210), infrared 700 nm donkey or goat anti-mouse IgG (1∶2000 for immunocytochemistry, 1∶10,000 for Western blotting; LI-COR Biosciences Cat. nos. 926-32222 and 926-32220), infrared 800 nm donkey or goat anti-rabbit IgG (1∶2000 for immunocytochemistry; 1∶10,000 for Western blotting; LI-COR Biosciences Cat. nos. 926-32213 and 926-32211), infrared 700 nm donkey anti-rabbit IgG (1∶2000 for immunocytochemistry; 1∶10,000 for Western blotting; LI-COR Biosciences Cat. no. 926-32223), and Alexa Fluor 488 nm goat anti-mouse IgG (1∶2000 for immunocytochemistry; Molecular Probes, Life Technologies, Eugene, OR, Cat. no. A11001).

### Primary Cortical Cultures

All efforts were made to minimize animal suffering and to reduce the number of animals sacrificed. Animal use was approved by the Duquesne University Institutional Animal Care and Use Committee (protocol number 1006-06), and carried out in accordance with the principles outlined in the *NIH Guide for the Care and Use of Laboratory Animals*. Allocortical and neocortical tissues were dissected with microforceps from the brains of postnatal day 1 or 2 Sprague Dawley rats (Charles River, Wilmington, MA) and incubated in 10 Units/mL papain (Sigma-Aldrich, Cat. no. P3125) for 30 min. Following a second incubation in 10% type II-O trypsin inhibitor (Sigma-Aldrich, Cat. no. T9253), cells were triturated in Basal Medium Eagle (Sigma-Aldrich, Cat. no. B1522) containing 10% bovine calf serum (BME/BCS, HyClone Thermo Scientific, Logan, UT, Cat. no. 2151,) supplemented with 35 mM glucose (Sigma-Aldrich, Cat. no. G8769), 1 mM L-glutamine (Gibco, Life Technologies, Cat. no. 25030-081), 50 Units/mL penicillin, and 50 µg/mL streptomycin (Gibco, Life Technologies, Cat. no. 15140-122). Dissociated cells were then seeded in Opti-MEM (Gibco, Life Technologies, Cat. no. 51985-034) supplemented with 20 mM glucose at a plating density of 100,000 cells/well in 96-well plates or 3 million cells/well in 6-well plates (Corning Costar, Corning Incorporated, Corning, NY). All plates were precoated with poly-D-lysine (1 µg/mL) and laminin (1.88 µg/mL, BD Biosciences), washed, and dried prior to seeding. After a 2-hour incubation of plated cells in Opti-MEM, a full media change was performed exchanging Opti-MEM for BME/BCS. Cells were then allowed a rest period of 2 days before initiation of toxin treatments.

### Validating Dissections of Neo- and Allocortex

In order to visualize the full anatomical extent of our microdissections, we dissected neocortex and allocortex from postnatal day 1 brains and then fixed the dissected brains in 4% paraformaldehyde. Fixed brains were cryoprotected in 30% sucrose in phosphate-buffered saline (PBS) and cut at a thickness of 50 µm in the sagittal plane on a microtome (Thermo Scientific Microm HM 450, Walldorf, Germany). Sections were stained with infrared DRAQ5 (1∶2000; BioStatus Limited, Leicestershire, UK) diluted in PBS with 0.3% Triton-X 100 for permeabilization. Washed and dried slides were imaged at 21 µm resolution on an Odyssey Infrared Imager (LI-COR, model number 9201-01).

### Toxin and Inhibitor Treatments

Because these cultures are initiated postnatally, the cortical neurons express high levels of punctate synaptic markers synaptophysin and synaptogyrin 3 already within a few days of culturing (R.K. Leak, unpublished observations). However, our postnatal cultures began to die 5–7 days after harvest. Treatments were therefore begun on day in vitro 2 (DIV2, 48 hours after plating). Cells were treated with 10× stocks of MG132, PSI, or H_2_O_2_ added to existing media along with 2.5 µM cytosine arabinofuranoside to suppress, but not eliminate, glial proliferation. Twenty-four hours later (DIV3) fresh media was exchanged for the old media (again with 2.5 µM cytosine arabinofuranoside). Multiple viability assays were then performed on DIV4 (see below). For inhibition of autophagy, 10× stocks of ammonium chloride (NH_4_Cl, 20 mM; Acros Organics, Somerset, NJ) or wortmannin (50 nM; Sigma-Aldrich) were applied in conjunction with MG132 on DIV2 and were freshly added to the media exchange on DIV3. N-acetyl cysteine (3 mM; Acros Organics) and rapamycin (0.025–3.2 µM; Research Products International, Mt. Prospect, IL) were applied with this same protocol.

### Immunocytochemistry and Viability Assays

Cell viability was assessed on DIV4, 48 hours after initiation of toxin treatments. In order to gain a broader view of cellular viability, we used two independent rapid, unbiased, and computerized assays, one for the neuronal marker MAP2 (see below) and one for ATP. ATP levels were assessed with a modification of the luciferase-based Cell Titer Glo assay (25 µl reagent in 50 µl media; Promega Inc., Madison, WI). Luminescence was measured within 15 minutes on a microplate reader (VICTOR^3^ 1420 multilabel counter; PerkinElmer, Waltham, MA).

For MAP2 immunocytochemistry, non-specific secondary binding was first minimized by blocking in a fish serum solution (Odyssey Block, LI-COR) diluted 1∶1 in PBS with 0.3% Triton-X 100. This was followed by serial incubations in primary antibodies (2 hr or overnight) and secondary antibodies (1 hr) in the Odyssey Block:PBS solution with PBS washes between steps. Infrared secondary antibody binding (700 and 800 nm) was visualized and quantified with Odyssey software (Version 3.0, LI-COR) on the Odyssey Infrared Imager, whereas visible-range secondary antibody (488 nm) binding was photographed on an epifluorescence microscope for higher resolution images (Advanced Microscopy Group, Model #AMF-4301-US, Bothell, WA). Hoechst nuclear staining (10 µg/ml Hoechst 33258, bisBenzimide) was completed in PBS with 0.3% Triton-X for 30 min.

### Western Immunoblots on *in vitro* and *in vivo* Lysates

Cultured cells were incubated for 10 min with Cell Lysis Buffer (Cell Signaling, Danvers, MA) supplemented with protease inhibitors (Sigma, Cat. no. P8340) and 10 mM sodium fluoride [84]. Scraped cells were sonicated for 20 pulses at 1 sec each (Misonix Inc. Model XL2020 Farmingdale, NY). Protein content in total cell lysates was quantified by the bicinchoninic acid method according to the manufacturer’s instructions (ThermoScientific). Equal amounts of protein (10–30 µg) were then separated on 10% polyacrylamide gels by standard sodium dodecyl sulfate gel electrophoresis and transferred to Immobilon-FL polyvinylidene fluoride membranes (EMD Millipore). Membranes were incubated in the Odyssey Block fish serum solution for 30–60 min at room temperature. All membranes were then incubated in primary antibodies in 50% Odyssey block diluted in PBS with 0.1% Tween overnight at 4°C on a shaker. The following day, blots were washed three times in PBS and incubated in the appropriate secondary antibody in the same diluent. Re-washed blots were then visualized and quantified on the Odyssey Infrared Imager. All proteins were expressed as a fraction of β-actin, α-tubulin, or GAPDH to control for differences in protein loading across treatment groups.

For *in vivo* immunoblotting, female Sprague-Dawley rats (Charles River) were sacrificed as a group at 2–4, 4–6, 8–9, 16–19, and 19–22 months of age. Tissue was weighed, sonicated in the abovementioned Cell Lysis Buffer (20 µL buffer per mg tissue), and standard immunoblotting as described above was then performed.

### Proteasome Activity

Proteasome activity was assessed 30 min after initiation of treatment with MG132 on DIV2 according to the manufacturer’s instructions (Cayman Chemical Company, Ann Arbor, MI, USA). In this assay, Suc-LLVY-AMC generates a fluorescent product when cleaved by the proteasome. Fluorescent intensity was read at 355 nm excitation and 460 nm emission (VICTOR^3^ 1420 multilabel counter). Proteasome inhibitors epoxomicin and the tea polyphenol epigallocatechin gallate were used as negative controls during the assay but did not lead to greater loss of proteasome activity than MG132. In addition to negative controls, Jurkat cell lysates were used as a positive control as this cell type exhibits high basal levels of proteasome activity. Nuclear DRAQ5 and MAP2 immunostaining on parallel plates was used to ensure equal protein content across treatment groups.

### Glutathione Assay

Glutathione levels were measured in neo- and allocortical tissue as a function of age. Dissected tissue was weighed and sonicated in PBS containing 2 mM EDTA (100 µL solution per mg tissue). The manufacturer’s instructions for the GSH-Glo assay for reduced glutathione were then followed (Promega Inc. Cat. no. V6911). This luminescent assay is based on conversion of a luciferin derivative into luciferin in the presence of glutathione and is catalyzed by glutathione-S-transferase. Data for each animal were collected in duplicate and expressed as relative luminescence units.

### Statistical Analyses

Data are presented as the mean and standard error of the mean from a minimum of three independently run experiments or from 4–5 animals per age group. With the exception of Western blotting, each *in vitro* experimental group was run in three microplate wells, from which data were pooled to yield one average value for that experiment. The two-tailed Student’s *t*-test was employed for two groups. Remaining data were analyzed by one- or two-way ANOVA followed by the Bonferroni *post hoc* correction (Graphpad Prism Version 5d). Differences were deemed significant when *p* ≤ 0.05.

## Results

### Characterization of Primary Neo- and Allocortical Cultures

**Figure 1:**
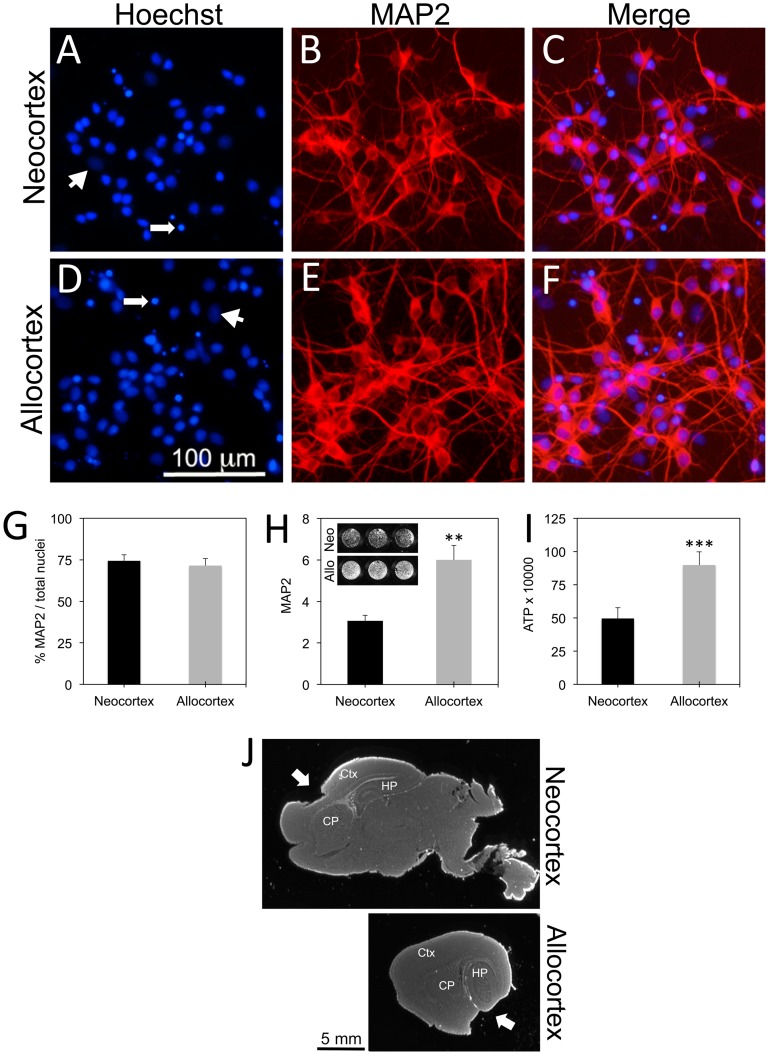
Characterization of *in vitro* model. **A–F:** Neocortical and allocortical tissue was microdissected from postnatal rat brains, dissociated, and plated in 96-well plates at 100,000 cells per well. Cells were treated with 2.5 µM cytosine arabinofuranoside to suppress, but not eliminate, glial proliferation on day in vitro 2 (DIV2). Plates were immunostained on DIV4 for the neuronal marker microtubule associated protein 2 (MAP2, shown in red). Nuclei were stained blue with Hoechst. Merged images reveal that the majority of the Hoechst-stained nuclei were neuronal. However, arrowheads point to large nuclei that were possibly glial. Long stemmed arrows point to condensed, potentially apoptotic nuclei that may have lost their neuronal phenotype. **G:** Blind cell counts revealed that nearly 75% of neocortical and allocortical Hoechst-positive cells expressed the MAP2 neuronal phenotype. **H and I:** Basal survival on DIV4 was measured by an infrared In-Cell Western assay for MAP2 and by the Cell Titer Glo assay for ATP. Both assays revealed that allocortex survived *in vitro* conditions at approximately twice the rate of neocortex. A grayscale inset of a representative infrared MAP2 stain of neo- versus allocortical neurons is included in **H.** Shown are the mean and standard error of the mean of at least 3 independent experiments. **p ≤ 0.01 or ***p ≤ 0.001 by the two tailed *t*-test. **J:** Brains were fixed following tissue dissections, cut in the sagittal plane, and stained for the infrared nuclear marker DRAQ5. Neocortical dissections (arrow) were centered in primary motor and sensory cortex (Ctx), dorsal to hippocampus (HP) and caudoputamen (CP). Allocortical dissections (arrow) were centered much more laterally in the brain ventral to hippocampus in the entorhinal and piriform cortices.

Immunostaining for the neuronal marker MAP2 and Hoechst-staining of nuclei on the dissociated primary cultures revealed that the majority of cells were MAP2 positive (see merged image in Figure 1, top). However, some large, flat nuclei (arrowheads, Figure 1A and D) and some condensed nuclei (arrows, Figure 1A, 1D) did not colocalize with MAP2. Some of the condensed and fragmented nuclei may represent apoptotic neurons that lost the MAP2 phenotype. In order to measure the percentage of the total culture that was neuronal, the number of MAP2 positive neurons and Hoechst-stained nuclei were both counted by a blind observer at 200× magnification in a 0.213 mm^2^ field of view (three fields per well). Expressing the number of MAP2 positive neurons as a fraction of total nuclei present revealed that nearly 75% of both neo- and allocortical cells expressed the MAP2 neuronal phenotype (Figure 1G). While this number may be considered low relative to early embryonic cultures, others have reported 50–80% neuronal purity in postnatal cultures treated with cytosine arabinofuranoside [85], [86]. Based on their staining for glial fibrillary acidic protein (data not shown), the remaining large, flat cells in these postnatal cultures may be astrocytic; astrocytes do not appear in large numbers until embryonic day 18 but they peak in the early neonatal period [87], [88]. It should be noted that glia play an important role in modifying disease processes [89]. Thus, the presence of at least some glia in postnatal cultures is closer to the natural state in the brain.

The photomicrographs in Figure 1 also reveal that allocortical cells survived the culturing process at a higher density than neocortical cells, even though both were initially plated at the same density of 100,000 cells per well in 96-well plates. This difference in basal survival was quantified by measuring MAP2 and ATP levels under normal, untreated conditions (Figure 1H and I).

Photomicrographs of our dissections are illustrated in Figure 1J. Postnatal day 1 rat brains were fixed in paraformaldehyde following dissections, cut in the sagittal plane, and stained with the infrared nuclear marker DRAQ5. The two regions harvested in this manner were centered in 1) primary motor and sensory fields dorsal to rostral striatum and rostral to hippocampus (part of frontal and parietal neocortex) or 2) piriform and entorhinal cortex ventral and lateral to the caudal hippocampus (all below the rhinal sulci and defined as part of allocortex) [87], [90]. Because these brain regions are at opposite poles of the brain, there was no risk of cross-contamination. Dissections that visibly included striatum or hippocampus were always discarded. However, we cannot claim that tiny numbers of hippocampal or striatal neurons were never included in our dissections because of the limitations of the dissecting microscope. This is the converse of small numbers of cortical neurons being included in hippocampal dissections in other studies. Nevertheless, by far the vast majority of plated neurons were from the primary sensory and motor cortices or the entorhinal and piriform cortices (Figure 1J).

### Validation of High-throughput Viability Assays

**Figure 2:**
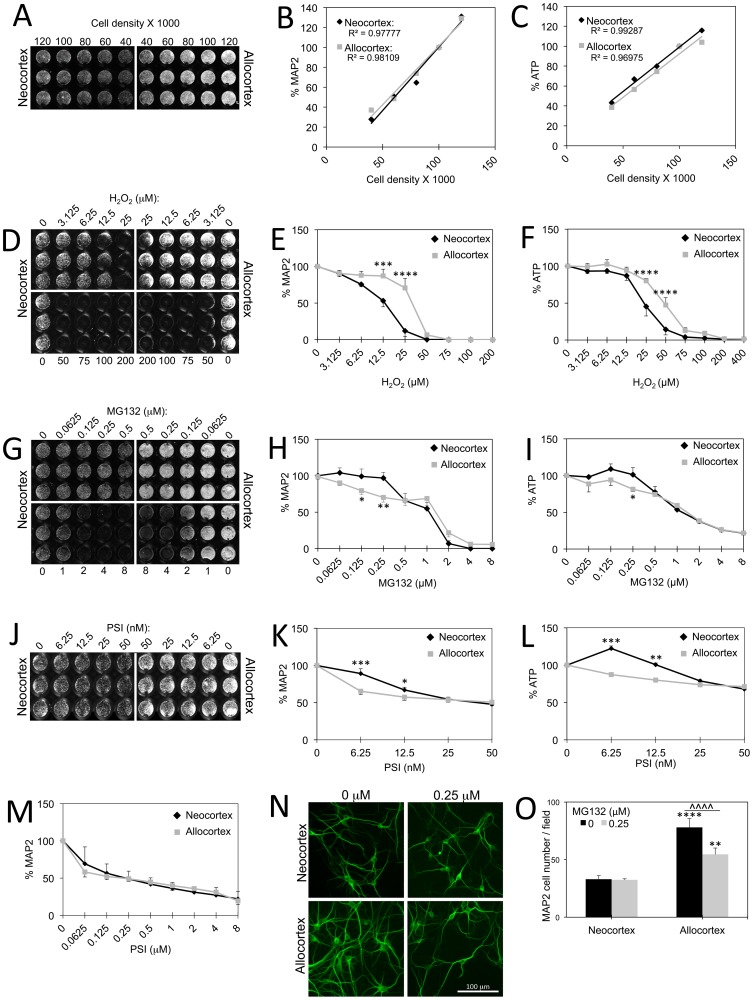
Differential vulnerability of neo- and allocortex to toxins. **A–C:** Neo- and allocortical cells were plated at cell densities that varied only by 20,000 cells per well (40K, 60K, 80K, 100K, and 120K cells per well). Plates were immunostained for MAP2 (**A and B**) or assayed for ATP (**C**). A linear correlation between signal and plating density was apparent with both assays at all densities except for ATP assessments of 120,000 allocortical cells. See Results section for *p* values. **D–F:** Neo- and allocortical cultures were treated with indicated concentrations of hydrogen peroxide. Allocortex was more resistant to oxidative stress by both MAP2 (**D and E**) and ATP assays (**F**). **G–I:** Neo- and allocortical cultures were treated with indicated concentrations of MG132 and assayed for MAP2 (**G and H**) and ATP (**I**). Both assays revealed that neocortex was less sensitive to low concentrations of this proteasome inhibitor. **J–M:** Neo- and allocortical cultures were treated with indicated concentrations of PSI and assayed for MAP2 (**J, K, and M**) and ATP (**L**). Allocortex was more vulnerable to low, but not high concentrations of PSI. Shown are the mean and standard error of the mean of at least 3 independent experiments. *p ≤ 0.05, **p ≤ 0.01, ***p ≤ 0.001, or ****p ≤ 0.0001 versus neocortex at the same MG132 concentration, Bonferroni *post hoc* correction following two-way ANOVA. **N:** Representative higher resolution photomontage of MAP2 immunostained neocortical and allocortical neurons treated with 0.25 µM MG132 or vehicle (dimethyl sulfoxide, DMSO). Results were quantified in Figure 2O. **O:** Neurons were counted by a blind observer at 200x magnification in a 0.213 mm^2^ field of view (three fields per well). Raw cell counts are shown to illustrate that there were more allocortical neurons under basal conditions. However, allocortical neurons were more vulnerable to 0.25 µM MG132. Shown are the mean and standard error of the mean of four independent experiments. **p ≤ 0.01, ****p ≤ 0.0001 versus neocortex; ^∧∧∧∧^ *p* ≤ 0.0001 MG132 versus vehicle (0 µM MG132); Bonferroni *post hoc* correction following two-way ANOVA.

We used two unbiased and computerized viability assays to measure cellular loss in a high-throughput and blind manner. One assay was for ATP levels (Cell Titer Glo), as a gross approximation of metabolic integrity. The other measurement was for neuronal MAP2 levels with an assay commonly called an In-Cell Western. This infrared MAP2 assay has already been validated in previous primary culture studies as an accurate means of assessing neuronal viability [91]. Nonetheless, in order to confirm the sensitivity and linearity of both assays, we plated neocortex and allocortex at cell densities that varied from 100,000 cells only by 20% (Figure 2A, B, and C; 40K, 60K, 80K, 100K, and 120K cells per well in 96-well plates). Signal strength was indeed sensitive to 20% changes in plating density for both MAP2 (Figure 2A and B) and ATP (Figure 2C) and was linearly correlated with cell number (for MAP2, neocortex R^2^ = 0.97777, *p* = 0.0014, allocortex R^2^ = 0.98109, *p* = 0.0011; for ATP, neocortex R^2^ = 0.99287, *p* = 0.0003, allocortex R^2^ = 0.96975, *p* = 0.0023). The infrared MAP2 assay was still sensitive to changes in allocortical and neocortical cell number at 120,000 cells per well, but the ATP assay could not distinguish between 100,000 and 120,000 cells per well in allocortex. As a result of these findings, we performed all our experiments at plating densities of 100,000 cells per well. Any decrease in cell number resulting from toxin treatments would then still be measurable for both neo- and allocortex.

### Toxicity of MG132 and Hydrogen Peroxide

As mentioned in the Introduction, Braak hypothesizes that allocortical cells may exhibit pathology earlier than neocortical cells because of reduced myelination [17]. This is speculated to result in higher demands on metabolic activity during neuronal firing, and as a consequence, greater oxidative stress in allocortex. In order to test the hypothesis that neocortex is more resistant to oxidative stress than allocortex, neocortical and allocortical cells were treated with the oxidative toxin H_2_O_2_ and assayed for MAP2 (Figure 2D and E) and ATP (Figure 2F). However, allocortical cells were far more resistant to H_2_O_2_ than neocortical cells by both measures. We conclude that treatment with H_2_O_2_ is unable to recapitulate the topography of protein aggregations in the human brain.

The Braak staging theory for both Alzheimer’s disease and Parkinson’s disease is not based on measures of oxidative stress *per se*. Strictly speaking, these topographical differences are dependent on immunohistochemical staining of misfolded α-synuclein and tau. These two regions therefore exhibit differential levels of proteotoxic stress. Thus, we treated allo- and neocortical cultures with the proteasome inhibitor MG132 to reduce the clearance of damaged proteins. At low concentrations of MG132, neocortex was more resistant than allocortex by both assays (Figure 2G – I). Furthermore, a significant interaction between MG132 and brain region was seen by two-way ANOVA of the MAP2 data, even when all concentrations of MG132 were included (*p* ≤ 0.05, F = 2.411). In other words, the impact of MG132 on viability depended upon whether the cultures were neo- or allocortical in origin. In order to verify the MG132 findings, we used a second inhibitor of the proteasome, PSI. Low concentrations of PSI elicited significantly more toxicity in allocortex by both assays (Figure 2J – L). ATP levels rose as a compensatory response to low concentrations of PSI, an adaptive phenomenon to mild stress known as hormesis [92]–[94]. Similar to MG132, higher concentrations of PSI did not elicit a differential response in neo- and allocortical cultures (Figure 2M). As with MG132, there was a significant interaction between brain region and PSI, verifying that the topographic origin of the cells determined the response to proteasome inhibition (*p* ≤ 0.001, F = 13.99 for MAP2, *p* ≤ 0.001, F = 15.07 for ATP). The proteasome inhibitor ALLN elicited similar responses as MG132 and PSI (neocortex significantly less vulnerable, but only to low concentrations; data not shown).

In order to verify the high-throughput viability results, we also counted MAP2 neurons by higher resolution microscopy following treatments with 0.25 µM MG132 (Figure 2N and O). This concentration was chosen based on the largest neo- vs allocortical difference in the MAP2 assay. Blind cell counts revealed significant neuronal loss in allocortex but none in neocortex, validating the high-throughput results. The neuron counts in Figure 2O are illustrated as raw data so that the higher basal survival in allocortex can also be appreciated.

### Interaction between Autophagy and the Ubiquitin-proteasome System

**Figure 3:**
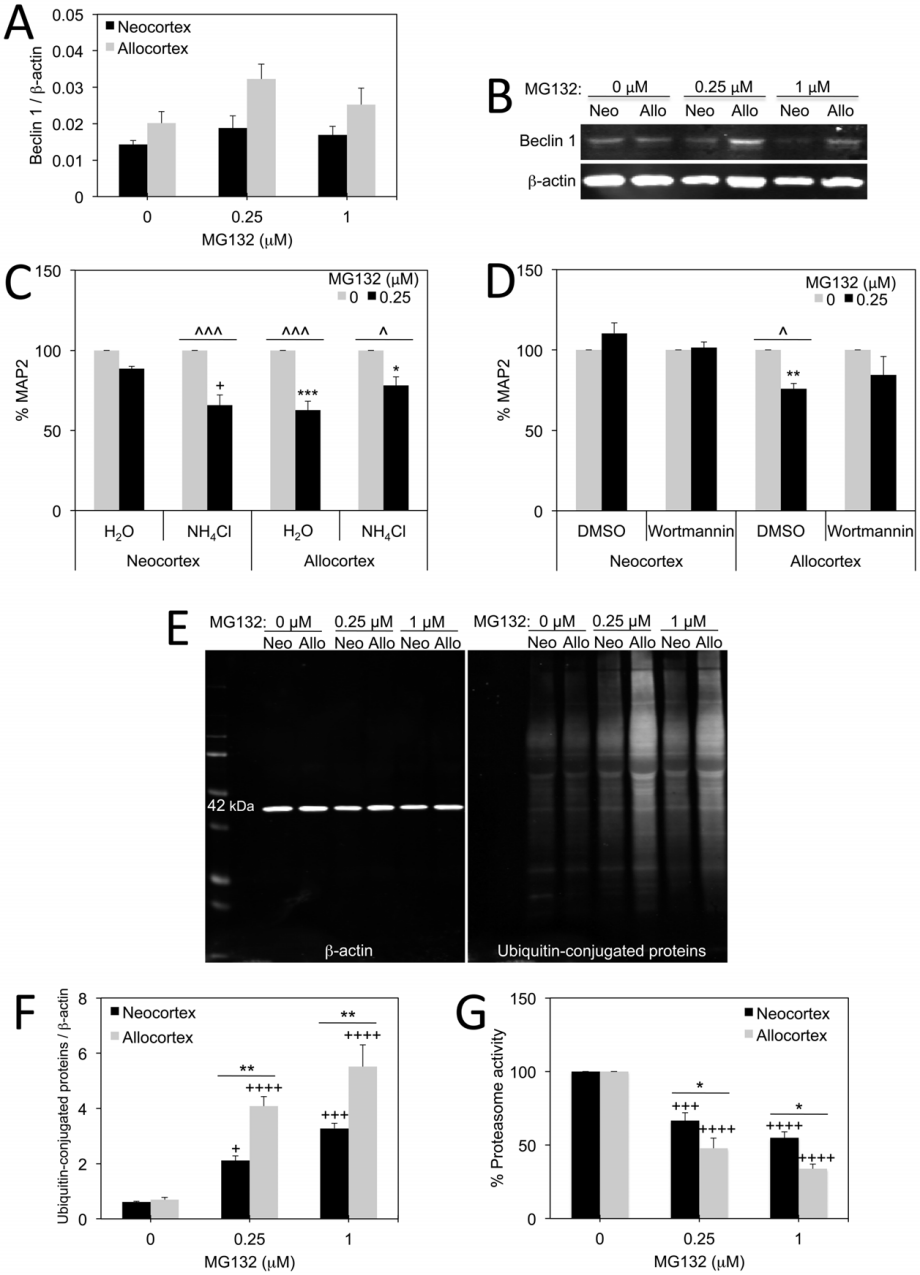
Involvement of autophagic defenses and ubiquitin-proteasome system. **A–B**: Infrared Western immunoblotting for macroautophagy-related molecule Beclin 1 following treatment of neo- and allocortical cultures with 0.25 and 1 µM MG132 is shown. β-actin was used as a loading control. **C–D**: Ammonium chloride (20 mM NH_4_Cl) was used to inhibit all forms of autophagy and wortmannin (50 nM) was used to inhibit macroautophagy in neo- and allocortical cultures subjected to vehicle or 0.25 µM MG132. Neocortical neurons became as vulnerable to MG132 as allocortical neurons in response to NH_4_Cl. Wortmannin failed to elicit an effect. Shown are the mean and standard error of the mean of at least 3 independent experiments. *p ≤ 0.05, **p ≤ 0.01, ***p ≤ 0.001 allocortex vs neocortex; ^∧^ *p* ≤ 0.05 or ^∧∧∧^ *p* ≤ 0.001 MG132 vs vehicle; +*p* ≤ 0.05 NH_4_Cl versus vehicle; Bonferroni *post hoc* correction following two-way ANOVA. **E–F:** Infrared Western immunoblotting for ubiquitin-conjugated proteins revealed that allocortex exhibited higher levels of this measure of proteotoxic stress in response to MG132. **G:** Proteasome activity was measured in the presence or absence of MG132 in a fluorogenic assay. Allocortical proteasomes were more inhibited by MG132 than those from neocortex. Shown are the mean and standard error of the mean of at least 3 independent experiments. For F and G, *p ≤ 0.05, **p ≤ 0.01 allocortex versus neocortex;+*p* ≤ 0.05,+++*p* ≤ 0.001,++++*p* ≤ 0.0001 MG132 versus vehicle (0 µM MG132); Bonferroni *post hoc* correction following two-way ANOVA.

The more vulnerable brain regions in neurodegenerative diseases such as allocortex are more likely to stain for lipofuscin pigments, or lipid residues of failed lysosomal digestion [18], [95]. Based on this observation, we speculated that allocortex may experience less autophagic clearance of cellular debris than neocortex. We probed for two proteins that are increased with macroautophagy, Beclin 1 and LC3BII [96]–[99] in neo- and allocortical cultures treated with 0.25 and 1 µM MG132. Although there were no significant changes in Beclin 1 by two-way ANOVA, there was a trend towards a rise in allocortical Beclin 1 at 0.25 µM MG132 (Figure 3A and B). In contrast, there was no change in LC3BII in any group (data not shown). Proteasome inhibition has been shown to raise cellular reliance on autophagy as an alternative means of degrading damaged proteins [100]–[103]. If neo- or allocortical cells rely on one of the three types of autophagy as a compensatory response to proteasome inhibition, inhibition of all forms of autophagy should further exacerbate MG132 toxicity. We therefore applied the pan-autophagy inhibitor NH_4_Cl and the macroautophagy inhibitor wortmannin to neocortical and allocortical cells in the presence or absence of MG132 (Figure 3C and D). NH_4_Cl is a weak base that accumulates in lysosomes and prevents all autophagic protease activity by neutralizing lysosomal pH [104]–[106]. Wortmannin is a PI3K inhibitor that inhibits macroautophagy but does not affect chaperone-mediated autophagy or microautophagy [106]–[108]. We chose wortmannin in place of the more commonly used 3-methyladenine because the latter PI3K inhibitor only inhibits macroautophagy under conditions of nutrient deprivation, whereas the 50 nM concentration of wortmannin suppresses autophagy regardless of nutrient status [108].

Our results with these two inhibitors reveal that the toxicity of 0.25 µM MG132 in neocortex was enhanced when autophagy was inhibited with NH_4_Cl (Figure 3C). In other words, neocortex became as vulnerable as allocortex with pan-inhibition of autophagy. Wortmannin did not affect the response to MG132 in either neo- or allocortex, suggesting that inhibition of macroautophagy had no effect (Figure 3D). When inhibiting macroautophagy has no impact, but overall autophagic inhibition with NH_4_Cl does exert an effect, chaperone-mediated autophagy or microautophagy are likely to be involved, a method of subtractive analysis that has been validated [106]. The NH_4_Cl data were therefore interpreted to preliminarily suggest that neocortex relied on chaperone-mediated or microautophagic defenses to clear unwanted proteins under conditions of proteotoxic stress, resulting in greater neocortical resilience to MG132. Inhibition of autophagy with neither NH_4_Cl nor wortmannin had an impact on allocortical vulnerability, suggesting that allocortex may not rely on any form of autophagy to battle MG132 toxicity. The wortmannin data are consistent with the LC3BII data and suggest that the trend towards a rise in allocortical Beclin 1 is insufficient to impact MG132 toxicity. Next we treated allocortical cells with the macroautophagy enhancer rapamycin in the presence or absence of 0.25 µM MG132 but found no evidence of any protection against MG132 toxicity (data not shown). The lack of protection with rapamycin is also consistent with the notion that allocortex does not rely on autophagy to survive proteasome inhibition. Finally, we repeatedly tried to immunostain neo- and allocortical cells for the chaperone-mediated autophagy marker lysosome-associated membrane protein type-2a (LAMP2a) or use Western immunoblotting to assess a potential rise in chaperone-mediated autophagy in neocortex, but found three LAMP2a antibodies to be non-specific in cortical cells (data not shown). Furthermore, because the NH_4_Cl effect size was small, we abandoned the autophagic line of inquiry and focused on the ubiquitin-proteasome system in subsequent experiments.

In order to test the hypothesis that the ubiquitin-proteasome system was more impaired in allocortex than neocortex, we used two independent measures of this system. First, we probed levels of ubiquitin-conjugated proteins by Western immunoblotting, as ubiquitin tails are conjugated to misfolded proteins to target them to the proteasome for degradation [109]–[11]. When proteasome inhibitors are applied, one therefore expects a rise in the number of misfolded, ubiquitin-conjugated proteins that cannot be degraded. We observed that MG132 significantly raised ubiquitin-conjugated protein levels in both neocortex and allocortex, suggesting that our proteasome inhibitor was exerting the desired effect (Figure 3E and F). However, the rise in ubiquitin-conjugated proteins was almost two fold higher in allocortical cells following application of either 0.25 or 1 µM MG132. These data indicate that the ubiquitin-proteasome system of degrading proteins may be more impaired in allocortex following a proteotoxic challenge.

As a second measure of the integrity of the ubiquitin-proteasome system, we directly measured activity of proteasome particles following an MG132 challenge. Consistent with the Western blotting experiments on ubiquitin-conjugated proteins, these experiments revealed that MG132 reduced proteasome activity in both neo- and allocortex following either 0.25 or 1 µM MG132, but that proteasome activity was even lower in allocortex at both concentrations (Figure 3G). In aggregate, these data support the hypothesis that this clearance system for degrading unwanted proteins is more easily compromised in allocortex.

### Neo/allocortical Differences in Stress-sensitive Proteins

**Figure 4:**
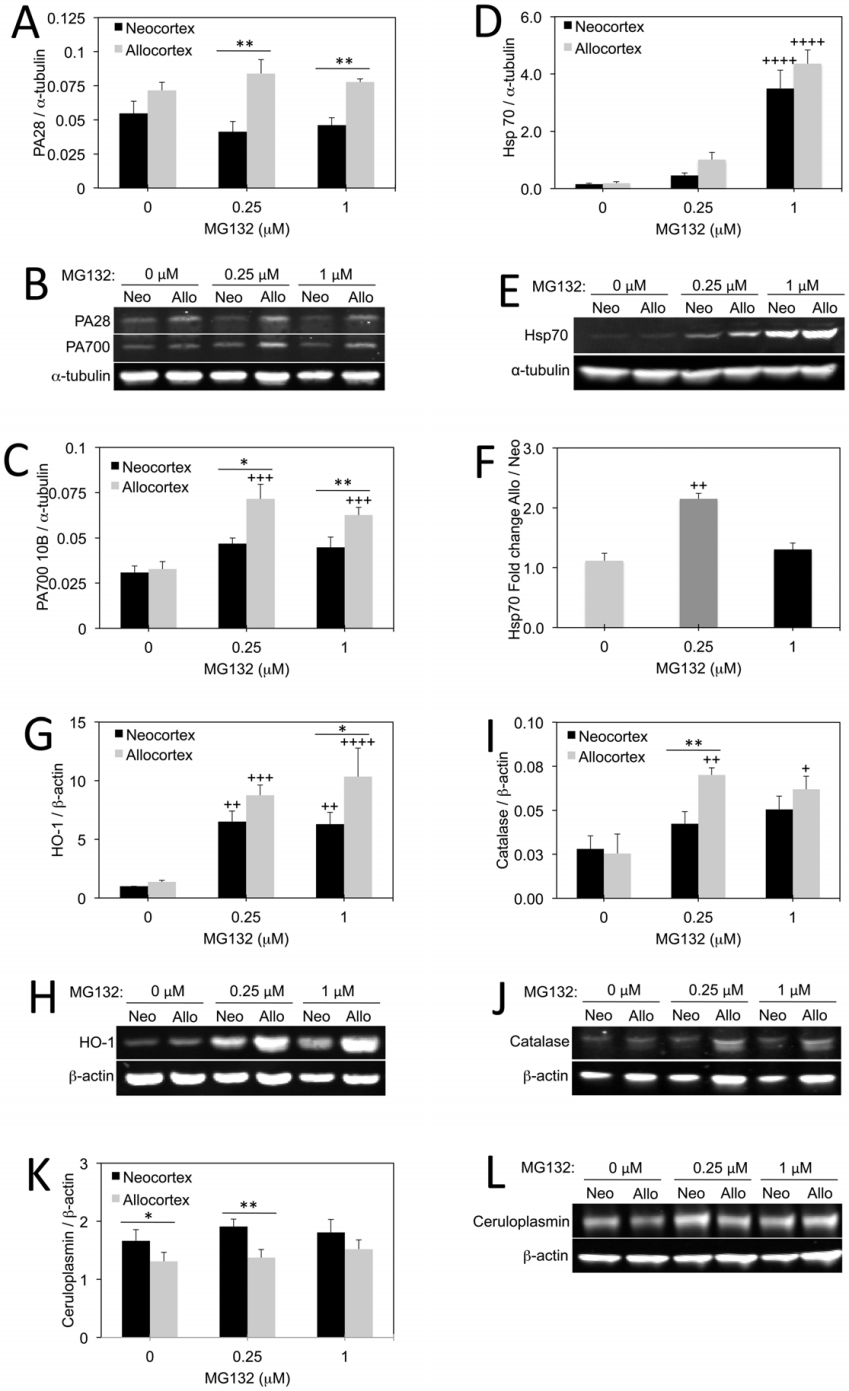
Protein changes in neo- and allocortex in response to proteasome inhibition. **A–G:** Western immunoblotting for PA28 (**A, B**), PA700 10B (**B, C**), heat shock protein 70 (Hsp70; **D, E, F**), heme oxygenase 1 (HO-1, also known as Hsp32; **G, H**), catalase (**I, J**), and ceruloplasmin (**K, L**) in neocortical and allocortical cultures treated with indicated concentrations of MG132. Shown are the mean and standard error of the mean of at least 3 independent experiments. *p ≤ 0.05, **p ≤ 0.01 allocortex versus neocortex;+*p* ≤ 0.05,++*p* ≤ 0.01,+++*p* ≤ 0.001,++++*p* ≤ 0.0001 MG132 versus vehicle; Bonferroni *post hoc* correction following one or two-way ANOVA.

Next we performed a series of immunoblotting experiments to quantify stress-sensitive protein changes following an MG132 challenge (Figure 4). First, we assessed levels of two proteasome activators, PA28 and PA700 [112], [113]. Binding of PA700 (the 19S regulator) to the 20S proteasome particle forms the activated 26S proteasome and binding of PA28 (the 11S activator) to the 20S proteasome activates the 20S particle. An ATP dependent interaction of PA700 with the catalytic core provides proteins with access to the core particle [114], [115]. In contrast, the 20S proteasome degrades short peptides and non-ubiquitinated proteins in an ATP independent manner [112], [113]. We honed in on these two proteasomal components because levels of PA28 are reduced in the superior frontal cortex (Brodmann area 9) in Parkinson’s disease whereas levels of PA700 are raised [56]. Our PA700 antibody recognizes the 42 kDa regulatory subunit 10B. We found no basal difference in levels of the PA700 or PA28 between allo- and neocortex in normal, untreated conditions (Figure 4A, B, and C). However, with the added stress of either 0.25 or 1 µM MG132, allocortex exhibited a rise in PA700. Furthermore, both PA28 and PA700 levels were higher in allocortex than neocortex following either 0.25 or 1 µM MG132. These data were interpreted to suggest that higher levels of proteotoxic stress in allocortical cells elicited a greater compensatory response in proteasome subunit expression than in neocortical cells. This interpretation of greater ubiquitin-proteasome stress in allocortex was supported by the previous data on ubiquitin-conjugated proteins and proteasome activity.

Because allocortical cells appeared to be particularly stress-sensitive, we then chose to assess levels of Hsp70, a well-studied folding chaperone that is induced under conditions of high proteotoxic stress [116]–[120]. As expected, we found that Hsp70 levels rose considerably in neocortex and allocortex in response to 1 µM MG132, supporting the notion that levels of this inducible protein are a biomarker of toxicity in our model (Figure 4D, E). When we expressed Hsp70 levels in allocortex as a fraction of levels in neocortex (Figure 4F), we found that there was an approximately two-fold difference between the two brain regions only at 0.25 µM MG132, the concentration that also elicited greater vulnerability in allocortex by the MAP2 and ATP assays. We interpreted these data to suggest that greater levels of proteotoxic stress in allocortex at 0.25 µM MG132 result in a higher Hsp70 chaperone response than neocortex. No such neo/allocortical difference was apparent at 1 µM MG132, the concentration that did not elicit differential vulnerability in the MAP2 and ATP assays. This meant that the difference in stress-induced Hsp70 levels correlated well with differences in cell and ATP loss.

We then examined stress-responsive Hsp32, also known as heme oxygenase 1. Heme oxygenase 1 is an inducible phase-2 enzyme that degrades toxic heme into by-products such as carbon monoxide and biliverdin [121]–[125]. Consistent with its inducible nature, we found that heme oxygenase 1 levels were considerably raised by both 0.25 µM and 1 µM MG132 (Figure 4G, H). The neo/allocortical difference was not quite significant at 0.25 µM MG132, but was significant at 1 µM MG132. Despite these compensatory responses in anti-apoptotic proteins such as Hsp70 and heme oxygenase 1, allocortex was not better protected than neocortex following either concentration of MG132. This reflects the greater vulnerability of allocortex to proteotoxicity and is discussed further below.

We next focused on antioxidant defense systems, as proteasome inhibition also raises levels of oxidative stress [126]–[128]. We probed for catalase, ceruloplasmin, and glutathione. Catalase is a ubiquitous enzyme that catalyzes the breakdown of hydrogen peroxide to water and oxygen. Catalase activity is higher in the frontal cortex and plasma of patients with Alzheimer’s disease relative to controls [129], [130]. However, catalase activity is decreased in the substantia nigra of Parkinson’s patients [131] and in the hippocampus of aged humans [132]. Proteasome inhibition with MG132 has been shown to upregulate catalase [133]–[135]. In support of some of these previous observations, we found catalase levels in allocortex to be raised by proteotoxic stress (Figure 4I, J). Neocortex did not exhibit a similar stress-induced rise in catalase. The difference between neo- and allocortex was only significant at 0.25 µM MG132. Thus, the differential catalase stress-response correlated with loss of viability in the MAP2 and ATP assays.

The copper chaperone ceruloplasmin is a ferroxidase that plays a protective role in the central nervous system [136], [137], [138]. Ceruloplasmin levels are high in the remaining neurons in Alzheimer’s disease, perhaps as a compensatory adaptation in surviving neurons [139]. Further, ceruloplasmin levels are higher in the substantia nigra in Parkinson’s disease [140]. Plasma ceruloplasmin also rises with stress in many human conditions [141]–[146]. We found that ceruloplasmin levels were higher in neocortex than allocortex both under basal conditions as well as following 0.25 µM MG132 (Figure 4K, L). At 1 µM MG132 there was no difference between neo- and allocortex. Ceruloplasmin did not show signs of being a stress-sensitive protein in our *in vitro* model. That is, MG132 did not raise ceruloplasmin levels at either concentration as it had the aforementioned proteins. Thus, the difference in ceruloplasmin between neo- and allocortex is not likely to reflect higher stress in neocortex than allocortex. This hypothesis is further supported by the observation that ceruloplasmin was higher in neocortex than allocortex even without MG132 on board.

### Involvement of Glutathione in Defense Against Proteotoxicity

**Figure 5:**
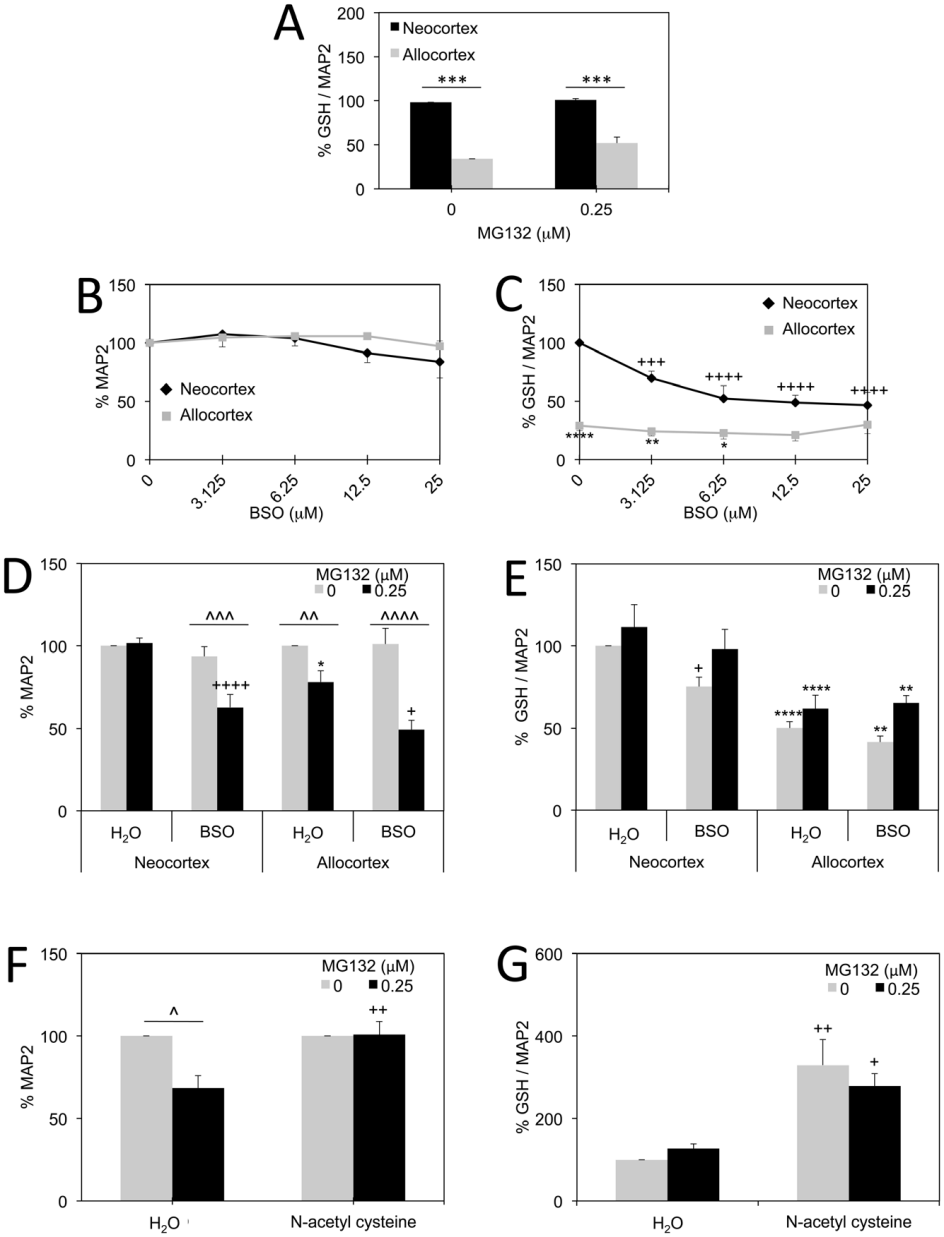
Role of glutathione in protection of neo- and allocortex against proteasome inhibition. **A:** Glutathione (GSH) levels as a function of total MAP2 levels in neocortex and allocortex in the presence or absence of MG132. Allocortical cells exhibited less glutathione than neocortical cells in both conditions *in vitro*. **B–C:** MAP2 levels (**B**) and glutathione levels (**C**) as a function of indicated concentrations of buthionine sulfoximine (BSO). Neocortical cells exhibited much greater glutathione loss with buthionine sulfoximine than allocortical cells. Buthionine sulfoximine was not lethal to neurons at the indicated concentrations. **D–E:** Neocortical and allocortical neurons were treated with vehicle or MG132 (0.25 µM) and vehicle or buthionine sulfoximine (12.5 µM). Both allocortical neurons and neocortical neurons were more vulnerable to combined treatment with buthionine sulfoximine and MG132 than either toxin alone (**D**). The glutathione assay revealed again that neocortical cells lost more glutathione with buthionine sulfoximine than allocortical cells, but that allocortical cells had overall less glutathione than neocortical cells in all four groups (**E**). **F–G**: N-acetyl cysteine (3 mM) completely prevented the toxicity of 0.25 µM MG132 in allocortical cultures (**F**) and considerably raised glutathione levels both with and without MG132 on board (**G**). Shown are the mean and standard error of the mean of at least 3 independent experiments. For all panels, *p ≤ 0.05, **p ≤ 0.01, ***p ≤ 0.001, or ****p ≤ 0.0001 allocortex versus neocortex; ^∧^ *p* ≤ 0.05, ^∧∧^ *p* ≤ 0.01, ^∧∧∧^ *p* ≤ 0.001, or ^∧∧∧∧^ *p* ≤ 0.0001 MG132 versus vehicle;+*p* ≤ 0.05,++*p* ≤ 0.01,+++*p* ≤ 0.001,++++*p* ≤ 0.0001 buthionine sulfoximine versus water or N-acetyl cysteine versus water; Bonferroni *post hoc* correction following two-way ANOVA.

Glutathione loss is one of earliest pathologies in both Alzheimer’s and Parkinson’s disease [59], [65]–[70]. We used a high-throughput infrared assay for glutathione (γ-L-Glutamyl-L-cysteinylglycine) in these studies. We previously validated that signal in this assay is lost with buthionine sulfoximine and rises with N-acetyl cysteine in many *in vitro* models, and that signal is also lost following preincubation of the antibody with reduced glutathione [135], [147]. Although this assay cannot distinguish between oxidized and reduced glutathione, most cellular glutathione is kept in the reduced state [148], [149]. This infrared assay revealed that allocortex cultures had considerably less glutathione than neocortex cultures under basal conditions as well as following treatment with 0.25 µM MG132 (Figure 5A). Similar to ceruloplasmin, glutathione did not robustly rise with MG132. Thus, the higher levels of ceruloplasmin and glutathione in neocortex are not biomarkers of greater proteotoxicity in neocortical cells and are positively correlated with relative resilience against MG132.

Following the observation of a striking difference in glutathione in allo- versus neocortex, we decreased glutathione levels with buthionine sulfoximine. First we treated both neo- and allocortex with concentrations of buthionine sulfoximine ranging from 3.125 to 25 µM and assayed for MAP2 to identify non-toxic concentrations (Figure 5B). MAP2 signal was not lost until 50 µM (data not shown). We found a significant loss of glutathione in neocortex beginning at 3.125 µM (Figure 5C). The smaller loss of glutathione in allocortex at 12.5 µM was not significant, perhaps because levels were already so low. Nonetheless, we determined the effect of 12.5 µM buthionine sulfoximine on neo- and allocortical vulnerability to MG132, with the understanding that this inhibitor worked far better on neocortex. Despite the difference in the efficacy of buthionine sulfoximine, we found that neocortex and allocortex were both more sensitive to proteasome inhibition in the presence of buthionine sulfoximine (Figure 5D). That is, buthionine sulfoximine and MG132 toxicities were synergistic. Importantly, neocortical cultures were as vulnerable to MG132 as allocortical cultures when neocortical glutathione synthesis was inhibited, suggesting that glutathione rendered neocortex more resilient to low concentrations of MG132. Assaying glutathione levels again verified that 12.5 µM buthionine sulfoximine elicited significant glutathione loss only in neocortex (Figure 5E). Furthermore, allocortex cultures exhibited severely reduced levels of glutathione relative to neocortex cultures in all four groups. Because a dual hit of buthionine sulfoximine and MG132 caused significant loss of MAP2 in allocortex without causing much glutathione loss, allocortex appeared to be exquisitely sensitive to the combination of the two poisons. In other words, buthionine sulfoximine decreased glutathione levels in neocortex more than allocortex and yet neocortex was not proportionately more vulnerable. This could reflect the basally higher levels of neocortical glutathione *in vitro*.

Because MAP2 levels in allocortex were so sensitive to buthionine sulfoximine when MG132 was applied, we investigated whether the glutathione precursor N-acetyl cysteine could raise glutathione levels in this vulnerable cell type and thereby prevent MG132 toxicity. As expected, this supplement greatly raised glutathione levels in allocortex and rendered allocortex as resilient as neocortex against the toxicity of 0.25 µM MG132 (Figure 5F and G).

### Neocortex and Allocortex Respond Differentially to Aging *in vivo*

**Figure 6:**
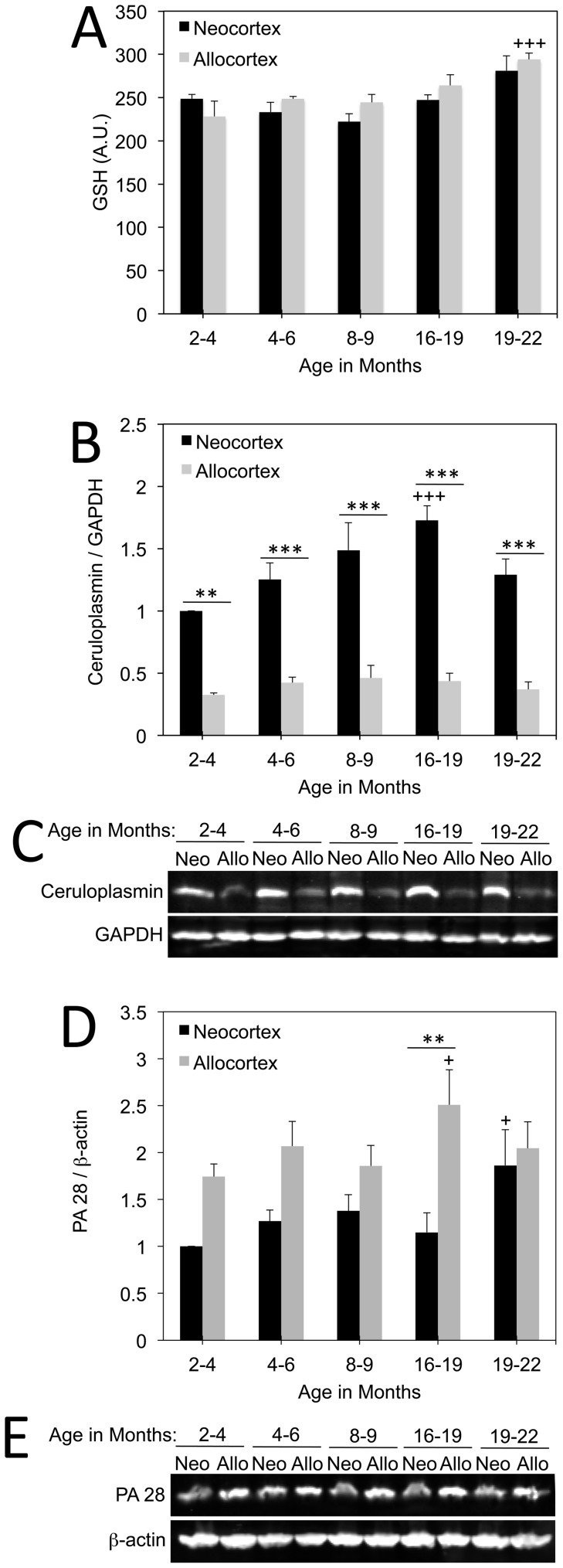
Impact of natural aging on neocortex and allocortex *in vivo*. **A:** Glutathione levels in whole tissue lysates of rat neo- and allocortex as a function of age. Allocortical glutathione levels were similar to those in neocortex but rose in an age-dependent manner and were significantly higher at 19–22 months of age relative to the youngest group (2–4 months). Neocortical glutathione did not change significantly as a function of age. **B–C:** Western immunoblotting for ceruloplasmin as a function of age and brain region. Neocortex had much more ceruloplasmin than allocortex at every age examined. Furthermore, neocortical ceruloplasmin rose in an age-dependent manner until rats were 16–19 months old. **D–E:** PA28 levels as a function of age in neo- and allocortex. PA28 levels rose in neocortex at 19–22 months and in allocortex at 16–19 months relative to the youngest age group. Allocortical PA28 levels were significantly higher than in neocortex at 16–19 months of age. Data are expressed as mean and standard error of the mean from 4–5 rats per group. For all panels, **p ≤ 0.01, ***p ≤ 0.001, allocortex versus neocortex;+*p* ≤ 0.05,+++*p* ≤ 0.001 versus levels in the 2–4 month old group; Bonferroni *post hoc* correction following two-way ANOVA.

As aging is a natural model of proteotoxicity [80]–[82] and glutathione levels were so much higher in postnatal neocortical cultures, we measured glutathione levels as a function of age in neo- and allocortical rat brain tissue (Figure 6A). Surprisingly, glutathione levels were not lower in allocortex in these whole tissue lysates at any adult age. However, glutathione levels rose slightly in allocortex as a function of age, with levels at 19–22 months being significantly higher than at 2–4 months of age. A linear regression analysis of glutathione as a function of age revealed a significant correlation between age and glutathione levels in allocortical tissue (*p* = 0.0136, R^2^ = 0.9014), but not in neocortical tissue (*p* = 0.2348, R^2^ = 0.4229).

Because ceruloplasmin was higher in neocortical cultures, we also probed ceruloplasmin levels in whole tissue lysates as a function of age (Figure 6B, C). Similar to the *in vitro* data, *in vivo* ceruloplasmin levels were higher in neocortex than allocortex at every age examined. Furthermore, neocortical ceruloplasmin levels rose linearly in an age-dependent manner until 16–19 months of age (*p* = 0.0140, R^2^ = 0.9723). Allocortical ceruloplasmin was not correlated with age (*p* = 0.3005, R^2^ = 0.4893).

Finally, in order to ascertain the stress on the proteasome, we measured ubiquitin-conjugated proteins as well as PA28 and PA700 levels as a function of age. Ubiquitin-conjugated proteins did not differ between neo- and allocortex and also did not rise with aging (data not shown). However, allocortical PA28 levels were higher in tissue from 16–19 month old animals than from 2–4 month old animals (Figure 6D, E). Neocortical PA28 was higher at 19–22 months of age than at 2–4 months of age. These responses may reflect age-related stress on the proteasome. A comparison of neo- and allocortical PA28 levels revealed that levels were significantly higher in allocortex at 16–19 months of age. Furthermore, the two-way ANOVA analysis revealed a significant impact of brain region on PA28 (*p* ≤ 0.001, F = 24.01). However, at 19–22 months of age, the difference between neo- and allocortex was lost, supporting the hypothesis that neocortex suffers from as much proteasome stress as allocortex toward the end of life.

## Discussion

The present study describes a novel, high-throughput model of topographic differences in vulnerability by microdissecting neocortex and allocortex from the postnatal rat brain and treating them in parallel fashion with toxins. The differential vulnerability of neo- and allocortex to low levels of proteotoxicity *in vitro* is analogous to their differential vulnerability to protein aggregations in Parkinson’s and Alzheimer’s diseases and several other neurodegenerative disorders [9], [18]–[22]. This is the first report that neocortical cultures are more resistant to proteotoxic, but not oxidative stress, than parallel cultures of allocortex. The neo/allocortical response to H_2_O_2_ suggests that allocortex has evolved remarkable defenses to combat exposure to oxidative stress. In contrast, the ubiquitin-proteasome system of allocortex was more impaired than that of neocortex. Allocortex was more vulnerable to proteasome inhibition despite far greater stress-induced rises in heat shock proteins, proteasome subunits, and catalase. Without these numerous compensatory adaptations, the allocortical and neocortical MG132 dose-response curves might have been shifted further apart than what was observed. We conclude that the ancient allocortex is not ill-prepared for either protein-misfolding or oxidative damage. In contrast to other markers, glutathione and ceruloplasmin levels were not robustly stress-sensitive and were basally higher in neocortical cultures. The neo/allocortical difference in glutathione levels *in vitro* was particularly striking but not reproduced with whole tissue lysates, as discussed further below with the *in vivo* results. Inhibiting either glutathione synthesis or autophagy rendered neocortex as vulnerable to low concentrations of MG132 as allocortex. However, allocortex was also vulnerable to inhibition of glutathione synthesis and enhancing glutathione synthesis with N-acetyl cysteine rendered allocortex as resilient to low concentrations of MG132 as neocortex. As a whole, our findings support the hypothesis that these subregions of the telencephalic pallium exhibit fundamental differences in their handling of cellular stress.

As with any study, there are experimental caveats in our *in vitro* system. For example, our initial experiments revealed higher levels of basal survival in allocortical cultures. We became concerned that varying cell densities at the time of treatment would lead to artifactual differences in neo/allocortical vulnerability to toxins. Specifically, we predicted that the improved basal survival in allocortex would lead to greater resilience because trophic factors released by the denser allocortex would be more concentrated in the media. This fear was allayed when we found opposite trends with an oxidative toxin and a proteasome inhibitor, suggesting that cell densities did not drive vulnerability in one direction or another. Importantly, recent data of ours reveal that neocortex survives just as well as allocortex in Neurobasal media and that the dose response curves look similar to those shown in the present study.

Another potential caveat of our and all other *in vitro* studies is the possibility that select phenotypic populations of neo- or allocortex survive the culturing process better than other, more vulnerable populations. This might enrich either neocortical or allocortical cultures in neurons with distinct vulnerabilities to MG132 and H_2_O_2_. On the other hand, if select neuronal populations were enriched in one or the other culture, we would not have expected to observe similar numbers of MAP2^+^ neurons as a fraction of total Hoechst^+^ cells (Figure 1G). Nonetheless, phenotypically distinct neuronal subgroups are likely to already be present *in vivo* and be reflected in our cultures even without any selective enrichment during the harvest and dissociation. Thus, future studies examining the GABA, glutamatergic, and other phenotypic subpopulations in the two brain regions and their potentially distinct vulnerabilities are warranted.

Our findings indicate that H_2_O_2_ treatment of these rodent cultures cannot be used to model the human topography of neo- and allocortical protein aggregations. Initially, this seems surprising, given that oxidative and protein-misfolding stress often propel each other. The H_2_O_2_ pattern is also unexpected considering that allocortical cultures exhibit lower basal levels of two antioxidants – ceruloplasmin and glutathione. Braak has noted that allocortex is less myelinated than neocortex and so might be subjected to higher metabolic demands and oxidative stress during neuronal firing [17]. Thus, the relative resilience of allocortex against oxidative damage may reflect an ancient defense against higher metabolic demands. Whatever the underlying reason, it is noteworthy that the relative resilience of neocortex in humans is largely based on histological observations of protein aggregates such as synuclein and tau, not on indices of oxidative stress *per se*. For example, the frontal neocortex exhibits oxidative stress even in incidental Lewy body disease [150]. In contrast to H_2_O_2_, proteotoxic stress from low levels of MG132 and PSI reproducibly elicited greater vulnerability in allocortex by two independent high-throughput viability assays, both of which were validated as linear and sensitive. Furthermore, manual counts revealed the same MG132 pattern as the high-throughput assays.

Autophagic and ubiquitin proteasome systems are thought to interact and collaborate in neuroprotection [151]. Inhibition of macroautophagy with wortmannin had no impact on either neo- or allocortex, suggesting that neither region relied on macroautophagy to battle MG132 toxicity. Conversely, raising allocortical macroautophagy with rapamycin failed to offer protection against MG132. Furthermore, inhibition of all forms of autophagy with NH_4_Cl had no impact on MG132 toxicity in allocortex. We conclude that allocortex does not rely on autophagy as an alternative method to clear cellular debris when the proteasome is inhibited. NH_4_Cl but not wortmannin worsened MG132 toxicity in neocortex, suggesting that neocortical cells may rely on chaperone-mediated or microautophagy when the proteasome is inhibited. This difference between neocortex and allocortex is consistent with postmortem observations that lipofuscin granules, a sign of failed lysosomal degradation, are more abundant in regions that exhibit denser protein inclusions [18], [95]. Although our findings warrant future examinations of rates of autophagy through pulse-chase experiments, it must be noted that the small fraction of cells that were lost following combined MG132 and NH_4_Cl treatment may render it exceedingly difficult to measure neo/allocortical differences in rates of autophagy. For these reasons and because three LAMP2a antibodies were non-specific in our system, we abandoned the autophagic line of inquiry and focused on the ubiquitin-proteasome system instead.

In addition to the viability assays, two independent assays verified that allocortex was subjected to greater proteotoxic stress than neocortex. First, ubiquitin-conjugated proteins rose more in allocortex than neocortex with both 0.25 and 1 µM MG132. The rise in ubiquitin-conjugated proteins suggested that MG132 was indeed effective in reducing the clearance of misfolded proteins in both allo- and neocortex. Second, we showed that allocortical proteasome activity was more depressed by MG132 at both 0.25 and 1 µM MG132. In retrospect one might then wonder why a difference in MAP2 and ATP viability was not also apparent at 1 µM MG132. One might speculate that allocortex mounts defensive adaptations in response to 1 µM MG132 that are not elicited with 0.25 µM MG132 and that are also not elicited by neocortex. Potential allocortical adaptations with 1 µM MG132 may then cancel some of the negative effects of enhanced inhibition of the proteasome at this concentration. An example of such an adaptation might be the rise in allocortical heme oxygenase 1 with 1 µM MG132.

At 0.25 µM MG132, allocortex also exhibited higher levels of Hsp70, catalase, PA700, and PA28 than neocortex. It is noteworthy that ubiquitin-conjugated protein levels were higher in allocortex despite elevated levels of the proteasome subunits PA700 and PA28. This pattern is consistent with previous studies also showing that proteasome subunit levels are elevated under conditions where there are more ubiquitin-conjugated proteins [152]. The hypothesis that these MG132-induced protein changes are defensive responses to proteotoxic stress warrants future investigations. It should be noted here that all our immunoblotting data are only correlative. Thus, future knockdown studies will be needed to determine if any of these proteins are mechanistically involved in conferring neuroprotection against proteotoxicity. As we did not assess levels of mRNA, we also cannot be certain that more proteins were synthesized. However, the considerable rise in several stress-sensitive proteins with MG132 is consistent with the hypothesis that levels were in proportion to the degree of proteotoxic damage. Finally, it is noteworthy that, even with stress-induced rises in anti-apoptotic folding chaperones and antioxidants, allocortex was still more vulnerable to lower concentrations of MG132 than neocortex. This also reflects greater vulnerability of allocortex to mild proteotoxic stress.

We assessed the mechanistic involvement of the ubiquitous tripeptide glutathione, present in millimolar concentrations in many cell types, where it effectively detoxifies reactive oxygen and nitrogen species [153]–[155]. Neocortex was rendered as vulnerable as allocortex to MG132 when glutathione synthesis was inhibited, suggesting that the higher levels of glutathione in neocortex were responsible for its relative resilience against low concentrations of MG132 *in vitro*. Buthionine sulfoximine did not lead to as much glutathione loss in allocortical cultures as it did in neocortical cultures and yet allocortex was still highly vulnerable to the combination of the two poisons, perhaps because glutathione levels were already low. These latter findings also underscore the relative vulnerability of allocortex to MG132. N-acetyl cysteine raised allocortical glutathione levels and made allocortex as resilient as neocortex to the toxicity of 0.25 µM MG132. Thus, N-acetyl cysteine seems to benefit MG132-treated allocortical cells with severely depressed glutathione levels.

Because of the pronounced difference between neo- and allocortical glutathione *in vitro*, we assessed levels of this ubiquitous thiol as a function of age *in vivo*. In contrast to the *in vitro* data, neo- and allocortex exhibited similar levels of glutathione across all ages. Whole tissue cortical lysates contain large numbers of glia, particularly astrocytes. Astrocytes are the major source of glutathione in the brain and high glutathione levels in this abundant cell type may have masked potential neuronal differences between neo- and allocortical glutathione. In contrast, the postnatal cultures were comprised mostly of neurons. Future flow cytometry studies that separate neurons from glia will be required to test the hypothesis that neuronal glutathione is indeed higher in neocortex *in vivo*. It should be noted that the glutathione antibody used *in vitro* does not discriminate between reduced and oxidized glutathione. However, most cellular glutathione is kept in the reduced state [148], [149] and signal in the assay is lost with preadsorption of the antibody with reduced glutathione. Finally, the difference we observed between neo- and allocortical glutathione *in vitro* may be purely a postnatal phenomenon or an artifact of the culturing conditions.

The rise in allocortical glutathione levels with age in whole tissue lysates might be a response to increasing levels of cellular stress because levels were highest in the oldest group and were well correlated with advancing age. After collecting the *in vivo* glutathione data, we performed a *post hoc t-*test on allocortical glutathione levels *in vitro* after treatment with MG132 and found a trend towards a small stress-induced rise (*p* = 0.07, see Figure 5A). These small changes in glutathione *in vitro* and *in vivo* are consistent with the notion that allocortex mounts compensatory adaptations against stress. Based on our *in vitro* data, we hypothesize that raising allocortical glutathione levels with N-acetyl cysteine would also attenuate proteotoxic stress *in vivo*. This could explain the success of N-acetyl cysteine in ameliorating cognitive deficits in Alzheimer’s patients [71].

Unlike the other defensive proteins, levels of the ferroxidase ceruloplasmin did not rise with MG132 in cultures. The higher levels of this protein in neocortex therefore do not reflect greater stress levels. Ceruloplasmin is relevant to neurodegeneration because loss of function ceruloplasmin mutations lead to extrapyramidal symptoms and parkinsonism associated with iron toxicity [156]–[160]. Furthermore, ceruloplasmin levels are low in the cerebrospinal fluid and serum of Parkinson’s patients while free copper levels are raised [161], [162]. Lower serum ceruloplasmin levels even correlate with younger disease onset [163]. There are also deficits in ceruloplasmin in Alzheimer’s disease [164]–[167]. Finally, ceruloplasmin deficient mice are more vulnerable to rotenone and stroke [136], [138]. We found that this protein was expressed at higher levels in neocortical cultures both under basal conditions as well as with 0.25 µM MG132. Our *in vivo* data revealed a more striking difference between neo- and allocortical ceruloplasmin levels than we found *in vitro*. Once again, we expect that the different glia-to-neuron ratio in the two model systems explains the discrepancy. Ceruloplasmin levels rose in neocortex until 16–19 months of age but not thereafter. These findings are consistent with a previous study that assayed developmental changes in ceruloplasmin until postnatal day 196 [168]. If higher cellular stress with aging raised ceruloplasmin levels in neocortex, we would have expected the highest levels at 19–22 months of age, but this was not the case. Alternatively, stress-induced defensive rises in ceruloplasmin may simply fail in senescent rats. No defensive rise in ceruloplasmin was observed with MG132. Thus, the age-related rise in neocortical ceruloplasmin may be a response to oxidative damage instead. If allocortex is also highly resistant to oxidative stress *in vivo*, this would be consistent with the lack of an age-related increase in allocortical ceruloplasmin.

Ubiquitin-conjugated proteins were similar in neocortex and allocortex *in vivo* and did not rise with aging. However, other studies have shown that aging decreases proteasome activity, including in the cortex [80]–[82]. Age-related stress on the proteasome was reflected in PA28 levels, which rose with aging in both neo- and allocortex. Furthermore, PA28 was higher in allocortex than neocortex, particularly in 16–19 month old animals. These findings mirror the *in vitro* data in neurons challenged with MG132, suggesting that allocortex may be subjected to greater proteasome-related stress *in vivo*. In the oldest animals examined, PA28 levels in neocortex and allocortex were similar because neocortical PA28 levels rose in this group, suggesting that the neocortex is also subjected to stress on the proteasome toward the end of life.

In conclusion, we found a large number of differences between allocortex and neocortex. These two regions responded quite differently to toxins *in vitro* and to aging *in vivo*. Our observations on differential responses to low concentrations of MG132 and PSI are consistent with clinical findings that human allocortex exhibits more proteotoxicity than neocortex. Enhanced vulnerability to low levels of chronic proteotoxicity in the diseased brain may lead to more cumulative damage in allocortex with aging. However, our findings also reveal greater allocortical defenses against oxidative toxicity and far more dynamic compensatory changes in response to proteotoxic stress. If similarly dramatic adaptations to proteotoxic stress occur in the human brain, one might speculate that this explains the generally slow and delayed nature of neurodegeneration.
