# Correction: Neocortex and Allocortex Respond Differentially to Cellular Stress *In Vitro* and Aging *In Vivo*

**DOI:** 10.1371/annotation/6e168e79-a961-4746-80f7-5626502197af

**Published:** 2013-10-17

**Authors:** Jessica M. Posimo, Amanda M. Titler, Hailey J. H. Choi, Ajay S. Unnithan, Rehana K. Leak

There were multiple errors in the Author Contributions statement. The Author Contributions statement should read: "Conceived and designed the experiments: RKL. Performed the experiments: JMP AMT. Analyzed the data: JMP AMT HJHC ASU. Wrote the paper: RKL." 

